# Assessing the feasibility, fidelity and acceptability of a behaviour change intervention to improve tractor safety on farms: protocol for the BeSafe tractor safety feasibility study

**DOI:** 10.1186/s40814-023-01319-w

**Published:** 2023-07-04

**Authors:** Aswathi Surendran, Jenny McSharry, David Meredith, John McNamara, Francis Bligh, Oonagh Meade, Denis O’Hora

**Affiliations:** 1 School of Psychology, University of Galway, University Road, Galway, H91 TK33 Ireland; 2 TEAGASC, Oak Park, Carlow, R93 XE12 Ireland

**Keywords:** Behaviour change intervention, Farm safety intervention, Blind-spots, Tractors, Peer-to-peer mentoring, Feasibility study, Farmers

## Abstract

**Background:**

In Ireland, the agriculture sector reports the highest number of fatalities even though farmers constitute only 6% of the working population. Tractor-related behaviours are implicated in 55% of all vehicle work-related fatalities and 25% of reported injuries, and many of these occur in farmyards. There is limited research on the feasibility and acceptability of behaviour change interventions to improve tractor safety. Target behaviours that promote safe operation in farmyards, determining and addressing blind spots of tractors, were identified, and an intervention was developed following the Behaviour Change Wheel Approach. The objective of the study is to examine the feasibility, fidelity and acceptability of a behaviour change intervention to enhance the safe operation of tractors in farmyards with a particular focus on tractor blind spots.

**Method:**

A single group feasibility study will be undertaken. Approximately 16 farmers from four major farm types will be recruited for the study between August and September 2022. The intervention involves an in-person demo session, facilitated discussion and personalised safety training procedure with safety goals. The study will collect data from participants at three time points: baseline (3–10 days prior to the intervention), during the intervention and at the follow-up session (7–30 days post-intervention). Quantitative data will be collected through a pre-intervention interview and feedback surveys. A pre- and post-intervention qualitative interview will also be conducted with the participants and will be supplemented with qualitative data from recruitment logs, observational memos and logs and feedback from recruiters. Evaluation of the feasibility, acceptability and fidelity of the intervention will be guided by a pre-determined feasibility checklist, fidelity framework and theoretical framework of acceptability, respectively. Interviews will be analysed using the content analysis.

**Discussion:**

The current study can determine the feasibility and fidelity of delivering a systematic, theoretically driven, tailored behaviour change intervention. It will also assess whether the intervention, its ingredients and delivery are acceptable to the farming population. This study will also inform the development of a future larger trial to test the effectiveness of the intervention.

**Trial registration:**

ISRCTN Identifier: ISRCTN22219089. Date applied 29 July 2022

**Supplementary Information:**

The online version contains supplementary material available at 10.1186/s40814-023-01319-w.

## Introduction

### Background

Farming is considered a dangerous occupation globally, accounting for a high number of occupational accidents and fatalities [1]. In Ireland, the farming industry makes up only 6% of the workforce [2]. Yet, for decades, the agricultural sector reported the highest number of fatal incidents of any other economic sector [3]. The Irish Agriculture and Food Development Authority (Teagasc) National Farm Survey (NFS) reported a 31% increase in farm accidents in the last decade in Ireland [4]. Farm machines, vehicles and livestock are linked to most of the serious incidents on farms [4, 5].

While there have been a variety of initiatives and interventions to tackle farm accidents and fatalities, a number of limitations with these studies have been identified. For example, intervention targets have been limited to adopting technical solutions or safety guidelines, and demographic factors like age and psychosocial factors have been often overlooked [6–9]. Farm safety research has also historically relied on safety education; however, recent reviews have raised questions about the effectiveness of education programmes to change farm operations and individual behaviours [1, 9].

Small-scale farms and family-run farms are regularly exempted from compliance with safety regulations, even though they work in an isolated environment with little to no oversight [10–12]. The lone-working nature of farming results in farmers’ being their own bosses; along with day-to-day farming responsibilities, they are often burdened with the identification of risks and the development and implementation of safety plans [9]. This often results in farmers prioritising the risk of financial failure over the risk of their own injuries by working long hours and taking unnecessary risks. These factors also act as barriers to adopting preventative safety behaviours such as routine maintenance activities [13]. Hence, a change in farm operation and farmers’ behaviour is required to improve the safety in farms.

Recent reviews indicate that safety interventions have been largely focused on the “Three Es”—“Education, Engineering and Enforcement” to prevent hazard exposures. Farm research literature clearly indicates that psychosocial factors play a crucial role and repeatedly indicates to focus on behavioural-based interventions targeting farmers’ attitudes and behaviours [14, 15]. The Medical Research Council (MRC) guidance for developing and evaluating complex interventions underscores the importance of integrating theory and available evidence in developing interventions [16]. Evidence emerging from public health research also indicates that behavioural theories and models can help to understand the determinants of behaviours, explain the behaviour change process to shape the behaviour and environment and identify potential behaviour change strategies to facilitate long-term changes [17–20]. Injury prevention literature also reports that successful strategies incorporated behavioural and environmental approaches [18, 19].

Analysis of risk factors and farm accidents indicates that demographic factors (e.g. age), farm characteristics, psychosocial factors (e.g. normalisation of farm accidents among farming communities and stress) and external factors (e.g. seasons and market pressures) influence farm safety [3, 21]. With an ageing farming population and age-related decline in physical and cognitive health, the older age (aged 65 +) group have been identified as having more injuries than any other age group [4, 8, 22]. A recent review by Nilsson [8] highlighted a lack of focus on older farmers in the intervention literature and that older farmers are less likely to participate and more likely to drop out of the safety initiatives. Previous studies have also indicated that the risk factors, behavioural practices and motivational factors differ across age groups [3, 13, 22]. Given that the pattern and impact of accidents vary across demographic groups, the needs of these vulnerable groups should be taken into account in the design of targeted intervention programmes in the future.

### Development of BeSafe Intervention

BeSafe project is a research programme funded by the Department of Agriculture, Food and the Marine (DAFM), Ireland, supported by the Irish Health and Safety Authority (HSA) and operated by Teagasc (the Irish state agency providing research, advisory and education in agriculture) to develop safety interventions to bring long-term changes to machine-related safety on farms. A lack of clear behavioural targets, limited integration of theory and limited reporting of the content of interventions have been identified as a major gap in existing farm safety literature [3, 15]. The BeSafe intervention was developed as a novel, theory-driven, tailored intervention that aims to address these gaps.

The Behaviour Change Wheel (BCW) framework was developed by synthesising 19 existing behaviour change intervention frameworks to provide a comprehensive and systematic approach for developing behaviour change interventions [19]. The BCW framework is based on the Capability-Opportunity-Motivation-Behaviour (COM-B) model, which suggests that for behaviour to change, an individual needs to have the capability, opportunity and motivation to engage in the target behaviour. These three components are interdependent and influence behaviour through multiple interacting factors. The COM-B model is linked to the BCW framework as it provides a theoretical understanding of behaviour change and helps to identify intervention functions, policy categories behaviour change techniques (BCTs) to support behaviour change. Intervention functions are the broad categories of strategies that can be used to change behaviour, such as education, training and environmental restructuring. BCTs are the observable, replicable and irreducible active ingredients of an intervention that can be used to change behaviour, such as goal setting, action planning and behavioural contract. This framework provides a structured approach for identifying the target population, target behaviour and the barriers and facilitators that need to be targeted by the intervention. This approach ensures that the intervention is tailored to the specific needs of the target population, is stakeholder-focused and is grounded in theory [23, 24]. Moreover, this approach allows for the evaluation of the feasibility, fidelity and acceptability of the active ingredients in a systematic and rigorous manner [19, 23].

As the first step of the intervention development, we systematically reviewed the interventions targeting machine-related accidents on farms [25]. Behavioural components present in these selected studies were then mapped to the BCW framework, and the behaviour change techniques to deliver these intervention functions were coded using the BCT taxonomy V1 [24]. Though the review identified nine intervention functions and twenty-one BCTs, the effectiveness of the BCTs was not assessed for various reasons, including the heterogeneity of the selected studies and missing information about the intervention components. Findings from the review encouraged the research team to create a tailored intervention that addresses vulnerable populations such as older farmers and is targeted at specific farm safety behaviours. Based on the review findings and Irish farm fatality reports, it was decided that the qualitative study would focus on the safe handling of tractor and quad-bike-related among older farmers. In the next phase, we conducted a qualitative study involving focus group discussions with older farmers (60 years and above). The focus group explored the barriers and facilitators to adopting safer tractor and quadbike-related behaviours. The findings were then thematically analysed and mapped to the COM-B domains. Participants have also suggested a few intervention strategies to address them, and the research team has mapped them to the BCW intervention functions and BCTs using the BCW framework and BCT taxonomy V1, respectively. In the next phase, we shared the findings from the review and focus groups with the panel members of the co-design workshops. Co-design workshops with experts and stakeholders identified the top target behaviours for the intervention, the potential behaviour techniques to address these behaviours and strategies for delivering these interventions. The subsequent meetings with the Teagasc representatives guided the selection and fine-tuning of the target behaviours (specific farm practices) and the intervention components. This exercise determined the usefulness and practicality of the intervention components and delivery strategies in the Irish farm context and ensured that they aligned with the Irish occupational safety approach. A detailed description of the intervention development is published elsewhere [26]. Intervention content was then mapped to the appropriate intervention functions of the BCW.

The BeSafe tractor safety intervention aims to equip farmers with knowledge, skills and resources to adopt preventive safety behaviours to address tractor-related blind spots on their farms and improve farm safety. To ensure the participation and feedback of older farmers, the study will ensure that at least 50% of the participants are aged above 60 years. The primary researcher, Teagasc representatives (FB and JM) and agricultural machine instructors conducted multiple mock demos and informally tested various aspects, timing and administration, of the intervention components to determine the timeline and resources for the intervention. The target behaviours (farm practices) targeted by the intervention are as follows:

- 1. Farmers to demonstrate blind spots of tractors to family members/co-workers on their farm
- 2. Farmers to mark the zone of visibility around their tractor in a parking area
- 3. Farmers are to complete a walk around the tractor each time before starting the tractor to check that there are no people or obstacles.

The BeSafe intervention is comprised of five intervention functions (Education, Training, Persuasion, Modelling and Enablement) and nine BCTs, selected to target barriers and facilitators associated with target behaviours (see Additional file 1 for the detailed breakdown of the intervention). The intervention involves an in-person peer-to-peer demonstration session, facilitated discussion and personalised safety training procedure with safety goals delivered (see Additional file 4 for an example safety training procedure form). The Template for Intervention DEscription and Replication (TIDieR) checklist [27] was used to describe the intervention (see Additional file 7 for TIDieR checklist for the BeSafe Programme).

Before evaluating the effectiveness of new interventions, it is recommended that intervention feasibility, fidelity and acceptability be assessed [16]. Complications that arise from the delivery, fidelity and acceptability can be effectively identified during a feasibility study, thus providing an opportunity to refine or modify before a larger effectiveness trial [16, 28]. Guided by Bowen et al.’s [29] framework for conducting feasibility studies, the current study will focus on the feasibility of implementing the intervention in an Irish farm setting, implementation of the intervention as intended (fidelity) and acceptability and adoption among recipients [29–31].

Findings from the study have the potential to inform the development of a full-scale randomised trial to evaluate its effectiveness in increasing safety practices on farms. Evidence emerging from the study also can inform future farm safety research on the feasibility and fidelity of implementing behaviour-change-based interventions and the acceptance of BCTs among farmers.

## Method/design

### Aim

This feasibility study protocol aims to outline the evaluation plans of the BeSafe intervention programme and provide a comprehensive description of the methods that will be used to assess its feasibility, fidelity and acceptability. The manuscript is structured in a way that describes the intervention and its theoretical underpinnings, the study design and methods, recruitment and participant selection criteria, proposed outcome measures and data analysis plans.

The objectives of the feasibility study are:

1. To assess the feasibility of recruiting the participants and delivering the intervention, which includes the recruitment, retention, adherence and completion of the intervention
2. To assess the fidelity of the design, training, delivery, receipt and enactment of the intervention
3. To evaluate the acceptability of the active components of the intervention among the participants
4. To identify participants’ perceived barriers and enablers to participating in, and completing, the intervention

### Study design

**Fig. 1:**
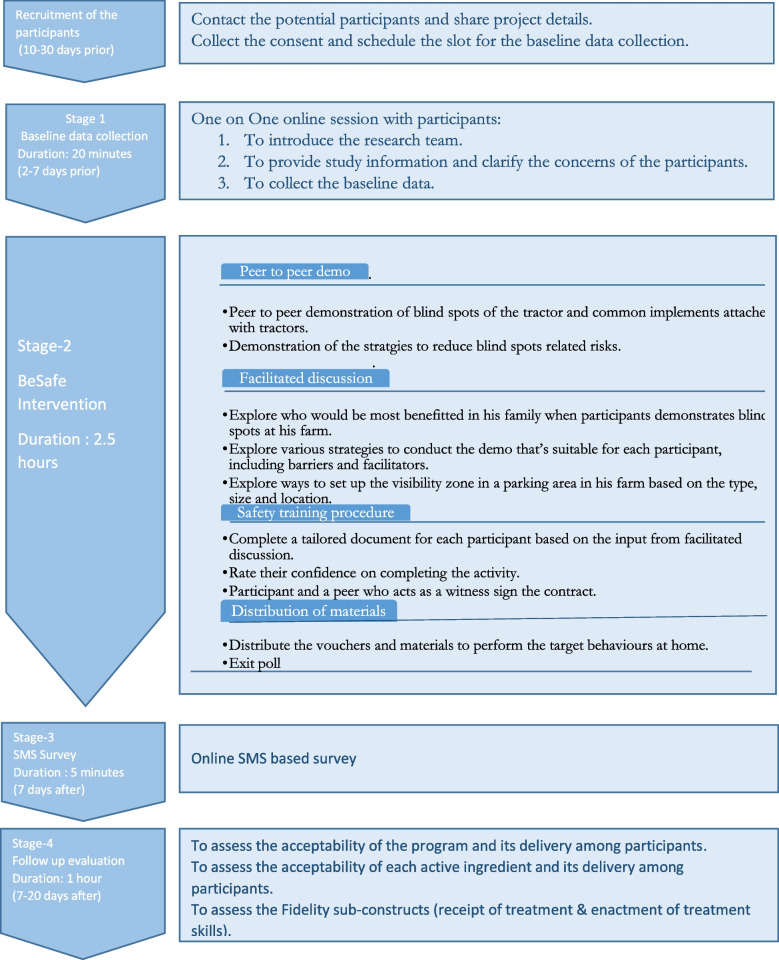
The timeline and components of the BeSafe intervention

This is a single-group feasibility trial. The study will use a combination of quantitative and qualitative methods to evaluate the intervention, including an exit survey and in-depth interviews with participants to gain insight into their experiences. Therefore, as per the guidance provided by the MRC framework for the feasibility trial, a mixed methods approach will be taken, as it will enable the integration of both quantitative and qualitative data to provide a more comprehensive understanding of the intervention [32–34]. The duration of the study will be 30–45 days based on the recommendation of stakeholders and the time limitation of the project. Baseline data collection will start at the beginning mid of August 2022, and the post-intervention interview sessions are estimated to be completed in approximately 2 months. The flow chart of the study is shown in Fig. 1. This protocol is reported according to Standard Protocol Items: Recommendations for Interventional Trials (SPIRIT) reporting guidelines (see Additional file 8 for SPIRIT Checklist) [35].

The participants’ demographic information will be collected during the pre-intervention interview, and descriptive statistics will be used to summarise the data. Short message service (SMS) surveys and exit surveys will explore the immediate feedback of the participants and the adoption of skills in their farm settings after the intervention. Post-intervention qualitative interviews will explore the feasibility of adopting the target behaviour, perceived acceptability and sub-constructs of fidelity. Content analysis [36] will be used to analyse the interview and survey data. Details of the data collected are provided in the ‘data collection’ section.

The *AgriDemo-F2F* (Agricultural Demonstrations and Farmer-to-Farmer Learning towards Sustainable Agriculture) is a European Commission-funded project that aims to enhance peer-to-peer learning within the farming community by building an interactive agridemo-hub community to promote practices and research-based farming solutions. The project is built on the principles of participatory and experiential learning, recognising that farmers learn best by seeing and doing [35, 37, 38]. The community provides guidelines and tools to design, implement and evaluate effective farmer-to-farmer learning approaches [35, 39–41]. As the current study involves peer-to-peer demonstration, we have incorporated participatory elements by following the recommendations and tools provided by the initiative. This will enhance participant engagement and comfort, and increase the effectiveness of the demonstration.

### Sample size

No formal sample size calculation was performed. There are several rules of thumb, ranging from 12 to 35 participants per group, for the pilot and feasibility studies [42, 43]. This study aims to recruit a minimum of 16 participants, just in excess of the minimum recommended sample size of 12, to compensate for the anticipated dropout. Other determining factors of the size were the availability of local resources and the maximum number of participants the research team could address in a session.

### Study setting

Teagasc is the semi-state authority in the Republic of Ireland responsible for research and development, training and advisory services in the agro-food sector. Teagasc personnel will assist the research team with:

- 1. Recruitment of the participants
- 2. Facilitation of in-person event
- 3. Arranging the demo site
- 4. Securing the site of intervention

All participants will be Irish farmers in contact with the Teagasc in the Republic of Ireland. The study will be conducted in selected Teagasc centres in the Republic of Ireland. To ensure the safety of the participants, the risks associated with each farm equipment that will be used in the intervention along with the preventive measures will be discussed during the session. Teagasc facilitators will be present on the demo site from the time of the preparation of the demo site to the conclusion of the event. One day before each demo, Teagasc safety officers will do a mandatory risk assessment and ensure that the demo site is safe for everyone. Safety checks will be done according to the HSA farm safety code of practice for risk assessment [44].

### Eligibility criteria

The average age of farmers in Ireland is 57, and fatal incidents involving farmers aged 65 or older account for 45% of farm safety incidents [3, 45]. In the previous interventions, the age range for “older farmers” varies between 55 [8, 46] and 65 [47], depending on the context of the study, but for this project, it was defined as those aged 60 and above. The intervention is primarily designed for older farmers, but younger farmers will be included in the trial to examine the generalizability of outcomes and tailor the intervention for different age groups.

### Inclusion criteria

- Currently working on farms part/full time
- Aged 18 years or older
- Have access to facilities and opportunities to share their learning from the study with someone who is not part of the study, such as a family member, neighbour or co-worker

### Exclusion criteria

- Participants who report emotional distress about farm incidents and safety
- Intend to participate in any other farm interventions during the study period
- Non-English speakers

### Recruitment strategy

Previous farm studies reported low participation and high attrition among older farmers [8, 22]. To ensure the participation of older farmers, at least half of the participants will be above 60 years of age. Details on the involvement of older farmers at the development stage of the study will be discussed in a separate publication.

The recruitment will be primarily carried out with the help of the Teagasc farm advisors using a purposive sampling strategy. At the beginning of the recruitment process, advisors will contact the eligible farmers to discuss the benefits of the research for farmers. To maximise the representation of different demographic groups, 50% of the participants will be aged above 60 years, and farmers from four major farm types, dairy, beef, sheep and tillage, will be recruited. The refusal rate and the reason for refusal cited by the farmers will be recorded. The research team will send the information sheet and consent form to the interested candidates. The contact details of the research team will be provided in case the participants have any questions. Once the participants sign the consent form and send it via post or email to the research team, the team will arrange a convenient time to conduct the pre-intervention phone call. During the call, before proceeding with baseline data collection, the researcher will talk the participant through the procedure to ensure they fully understand what they are consenting to and is comfortable talking about fatal incidents and safety. This will be then followed by demographic data collection (see Additional file 3 for an example topic guide).

To ensure maximum retention, participants will be offered vouchers at the completion of each stage of the study. Every participant who completes all stages of the study will receive vouchers worth 100 euros by the end of the study. The introduction session is also intended to create a rapport between the research team and the participants.

### Withdrawal of study participants

Participants will be given a minimum of 2 weeks to make the decision about their participation and every opportunity to clarify any enquiries they have related to the project. Participants will be informed at every stage of the programme that they can withdraw from the study without any penalty and without giving any reason. Participants may get re-allocated to a different session or removed from the study by the researcher if they exhibit any symptoms or test positive for COVID-19 infection. The data collected from that participant up to that point will be considered for evaluation unless specifically requested by the participant to delete it.

### Procedure

Participation in the study includes four stages. The stages and their timeline is as shown in the flow diagram. Once the participant expresses interest and provides consent, the primary author will contact them to collect the baseline data and confirm their attendance at the in-person event. The intervention involves a half-day in-person event and identical demo sessions with a maximum of six participants in each session will be conducted. The maximum number of participants per session was determined per the recommendation of agridemo guidelines [35, 48] and feedback from the informal demo session. Participants can choose to participate in any one of the sessions based on their convenience. At the end of the intervention, participants will be asked to complete an exit survey to share their feedback about the intervention (see Additional file 5 for an example exit survey). The primary author will send an SMS survey after 7 days to monitor the participant’s progress in the adoption of safety goals (target behaviours). All participants will be invited to take part in an online interview within 20 days of the intervention to assess the perceived acceptability of the intervention among participants.

#### Materials

The participants will be provided with all materials to implement the safety strategies demonstrated and discussed in the intervention session.

### Data collection

#### Baseline assessments

The primary author will collect demographic data and the safety devices currently used by the participant during the introductory session (stage 1) using a pre-prepared questionnaire (see Additional file 1 for an example questionnaire).

#### Follow-up assessments

Immediately after completing the intervention session, participants will be requested to fill out an anonymous exit survey. An exit survey is a modified version of the feedback form provided as a part of the EU H2020 AgriDemo [35, 40, 48]. The objective of this survey is to gather participants’ immediate feedback on the acceptability of intervention components. This information may also help to gain the feedback of participants who may drop out before the post-intervention interview and is expected to support the researchers in refining the interview guide for the qualitative interview. The convenient time to receive the SMS survey and set up the interview would also be collected at the end of the session.

The SMS survey is intended to remind and track the progress made by the participants on the adoption of the safety goals at their farms. The follow-up evaluation interview will be a 1-h semi-structured online interview to explore perceived acceptability, measure receipt of treatment and enactment of skills (see Additional file 6 for an example topic guide). While the SMS survey measures the progress on the completion of safety goals (home-based tasks), the interview will further explore their experience with performing the tasks at home and if they faced any barriers to completing the activities.

### Study outcomes and measures

A summary of the objective, measures and tools to assess the outcomes are provided in the table.

#### Feasibility outcomes

Consideration of feasibility criteria was guided by the agriculture literature and feasibility study guidelines [29, 49]. Feasibility will be measured based on the criteria detailed in Table 1.

**Table 1:** Data collection and evaluation plan

**Objective**	**Indicators and outcome measures**	**Data collection method**	**Measure of success**
**Feasibility Checklist**			
**Feasibility**	Recruitment rate: % of participants recruited/time	Recruitment report by the Teagasc.	**Recruitment:** Successfully recruit a minimum two farmers from each of the four farm types: dairy, beef, sheep and tillage
	Retention and follow-up rates: % of participants completed the stage 1, stage 2, stage 3, stage 4 (Fig. 1)	Audio recording of the in-person sessions Intervention checklist (Additional file 1)	
	Time required to recruit target sample size: 16 participants recruited/time	Recruitment report by the Teagasc	
	Adherence rates: % of participants completed the stage 1, stage 2, stage 3, stage 4	Field notes and Memos from the in-person sessions	**Retention rate:** 80% of the participants complete the in-person session & A minimum of 12 participants complete follow-up interviews
	Rate of completion of the intervention: % of intervention activities completed by the participants	Field notes and Memos from the in-person sessions	
		SMS Survey	
	Representation of farm population: % of participants recruited from four farm types/total participants	Pre-intervention interview data	
	Barriers & facilitators to set up the in-person event: Factors identified by the facilitators	Recruitment report by the Teagasc Audio recording of the in-person sessions Field notes from the in-person sessions	**Programme structure:** At least 80% of the participant rate the programme structure and content as satisfactory
	Is the structure of the programme (length of the event, structure and content of the intervention) realistic, clear and reasonable for the participants?: Average score for each of the responses in the exit survey	Exit survey	
		Post-intervention interview	
	The ability of the participants to carry out the target behaviours (safety behaviours) addressed in the study: Participants’ feedback about their experience	Safety training procedure Exit survey Post-intervention interview	
**Fidelity Checklist** (1)			
**Fidelity: **Intervention Design	1—Provide information about treatment dose in the intervention condition: Detailed information about the intervention is presented in the intervention checklist, such as: a. Length of session b. Number of sessions c. Content of each session d. Duration of contact over time	Intervention checklist	**Intervention report**: Detailed report on the development of the intervention, content of the intervention and BCTs included in it using TiDier checklist
	2 Theoretical model upon which the intervention is based is clearly articulated in the methodology paper and includes the following information: a. The active ingredients are specified and incorporated into the intervention b. Use of experts or protocol review group to determine whether the intervention protocol reflects the underlying theoretical model or clinical guidelines c. Plan to ensure that the measures reflect the hypothesised theoretical constructs/mechanisms of action	A manuscript describing the development of BeSafe Intervention	**Trial registration:** Register the study on ISRCTN registry
	3 Potential confounders that limit the ability to make conclusions at the end of the trial are identified?	Post-intervention interview	
	4 Plan to address possible setbacks in implementation (i.e. back-up systems or providers)	Risk assessment document	
**Fidelity: **Treatment providers*	1 Description of how providers will be trained (manual of training procedures)	Intervention manual	**Intervention manual:** Complete and upload the intervention manual in a public repository
**Fidelity: **Treatment delivery	1 Method to ensure that the content of the intervention is delivered as specified (content, dose, process): Number of intervention components delivered as intended/Number of intervention components expected to be delivered	Audio recording Intervention checklist Field notes	**Delivery**: A minimum of 80% of the intervention components presented in the intervention checklist are delivered.
	2 Assessment of non-specific treatment effects: Participants report on the experience of the programme vs expected outcome	Post-intervention interview	
	5 Use of Intervention manual	Project information sheet	
	6 There is a plan for the assessment of whether or not the active ingredients were delivered: Number of active ingredients delivered as intended/Number of active ingredients present in the BeSafe intervention	Audio recording Intervention checklist Field notes	
	7 There is a plan for the assessment of whether or not proscribed components were delivered. (e.g. components that are unnecessary or unhelpful)	Audio recording Intervention checklist Field notes	
	8 There is a plan for how will contamination between conditions be prevented	N/A	
	9 There is an a priori specification of treatment fidelity (e.g. providers adhere to delivering > 80% of components): Reference to ‘measure of success’ column	Data collection and evaluation plan	
**Fidelity: **Receipt of Treatment	1 There is an assessment of the degree to which participants understood the intervention: Participants’ report on their understanding of the intervention skills	Safety training procedure Exit poll Post-intervention interview	**Receipt:** A minimum of 60% of the participants complete the target behaviours at home
	2 There are specifications of strategies that will be used to improve participant comprehension of the intervention: Completion of tailored plan using the safety training procedure document an participants’ feedback	Safety training procedure Exit poll Post-intervention interview	
	3 The participants’ ability to perform the intervention skills will be assessed during the intervention period: Self-report on the confidence in completing the target behaviours and participants’ feedback	Safety training procedure Exit poll Post-intervention interview	
	4 A strategy will be used to improve subject performance of intervention skills during the intervention period: Hands-on practice session on demonstration, completion of tailored plan using the safety training procedure and participants’ feedback	Peer to peer demo Safety training procedure	
	5 Multicultural factors considered in the development and delivery of the intervention (e.g. provided in native language; protocol is consistent with the values of the target group)	N/A	
**Fidelity: **Enactment of Treatment Skills	1 Participant performance of the intervention skills will be assessed in settings in which the intervention might be applied: Participants’ self-report on the completion rate of safety goals (target behaviours)	SMS Survey Post-intervention interview	
	2 A strategy will be used to assess performance of the intervention skills in settings in which the intervention might be applied: Participants’ reported experience on completing the safety goals at home	Post-intervention interview	
**Acceptability checklist**			
**Retrospective acceptability**	1 Affective attitude (How did the participant feel about the programme): Average satisfaction score for the programme in the exit survey	Safety training procedure Exit survey Follow-up evaluation interview	A minimum of 80% of the participants rate the programme on exit survey as “Agree” or “Strongly agree” A minimum of 80% of the participants rate their confidence in completing safety goals at least 7 out of 10
	2 Burden (What did the participants say about the structure and ease to understand): Average satisfaction score for the programme structure in the exit survey and participants’ feedback		
	3 Ethicality (To what extent the strategy helped in performing the farm tasks): Average satisfaction score for the programme and topics covered in the programme in the exit survey		
	4 Intervention coherence (How did the participant feel in terms of understanding the tasks and performing them by himself): Participants’ report on what they learned during the programme		Evidence for perceived benefits and effectiveness of the intervention in the follow up interview.
	5 Opportunity costs (What were the benefits the participants perceived): Participants’ feedback		
	6 Perceived effectiveness (To what extent did the participant feel that strategy was effective): Participants’ report on what they learned during the programme		
	7.Self-efficacy (How confident and comfortable was the participant at performing the task): Average confidence score for each of the target behaviours in the safety training procedure document		

*Treatment is delivered by the research team. Hence no training sessions required

These criteria will be evaluated through an intervention checklist, direct observation, audio recording of the in-person intervention sessions and reported experience of the facilitators.

Additionally, the current study will recruit farmers who have opportunities to demonstrate the learnings from the intervention to a non-participant person. During the feasibility evaluation, the authors will examine the report from the recruiters to determine whether this criterion came up as a barrier to recruitment or retention. This is especially relevant as farming in Ireland is generally small scale, with an average farm size of fewer than 14 ha, and farmers regularly meet through discussion groups or similar events [50, 51]. Peer-to-peer demonstration and facilitated discussion present a unique opportunity for knowledge sharing and learning within the community.

#### Fidelity of the intervention

Existing reviews indicate that the fidelity of the interventions is not often measured, reported or accounted for in the research [52, 53]. Measuring fidelity can not only ensure that core components are delivered as intended but also measure how it is delivered, the quality of the delivery and participants’ engagement [52]. Following international guidance, five components of fidelity, treatment design (the degree to which the delivery adhered to the behaviour change theory that informed the design), provider training (adequacy of the training and resources to deliver intervention), intervention delivery (the degree to which the intervention components were delivered as intended), intervention receipt (the degree to which the recipients demonstrate the ability to understand and perform the safety practices), and enactment (the degree to which recipients execute the practices in their own farms), will be assessed [54, 55]. By systematically assessing different aspects of the fidelity of the intervention, researchers can identify where there might be gaps or discrepancies between what was intended and what is actually delivered. Moreover, measuring receipt helps to ensure that the intervention was delivered as intended and to the correct audience. Enactment, on the other hand, provides an accurate representation of participants’ actual performance of intervention skills or implementation of the core intervention components in the intended situation. This information is important for improving the intervention’s replicability and generalizability [54].

In order to measure how well the intervention is delivered as intended, the fidelity of design, training and delivery will be assessed by the primary author using a pre-established checklist. To evaluate the participants’ understanding of the intervention components and application of the intervention skills in their day-to-day life, the fidelity of receipt and enactment will be explored in the follow-up assessment [54, 56].

Since the intervention is delivered by the research team, no training is required. However, the authors will be publishing an intervention manual for future studies, which will address the resources, skills and training required.

#### Acceptability of the intervention

A review of farm safety interventions observed that even when the researchers report the intervention and its delivery, they provide little to no information on perceived acceptability for the programme among participants [57]. Given that perceived acceptability reflects the extent to which the intervention was perceived as appropriate, the successful adoption of the intervention relies on it [58, 59].

During the intervention development phase, the study utilised the BCW framework to identify BCTs for inclusion in the intervention. As part of this process, the APEASE (Acceptability, Practicability, Effectiveness, Affordability, Spill-over effects, and Equity) criteria were utilised to ensure that the selected BCTs met the necessary criteria of being acceptable, practical and effective [23]. However, to evaluate the acceptability of the intervention and its components among participants, the study will use the Theoretical Framework of Acceptability (TFA) framework. The TFA framework is a comprehensive and flexible framework that provides a structured approach to understanding the acceptability of interventions. It enables researchers to explore how participants feel about the intervention and why they feel that way. The study will explore the seven component constructs of TFA: Affective attitude, Burden, Ethicality, Intervention coherence, Opportunity costs, Perceived effectiveness and Self-efficacy [59]. By breaking down the concept of acceptability into several domains, the TFA framework allows researchers to identify which aspects of the intervention are most important to participants and where there might be issues with acceptability. This information is essential for tailoring the intervention to meet the needs and preferences of the target population, which can increase its uptake and effectiveness.

The follow-up assessment involves an in-depth interview that will explore the participant’s retrospective perceptions of the acceptability of the intervention, the expectations and experiences of the intervention, and its key components.

### Data analysis

As per the pilot and feasibility studies guidelines, quantitative data analysis will be descriptive [60, 61]. The demographic characteristic of participants and the use of safety devices on their farms will be summarised using descriptive analysis. Qualitative data will be audio-recorded and transcribed verbatim using a third-party transcription service. Transcribed data, along with survey data, documented field notes and memos, will be managed using MaxQDA 2020 [62] and analysed by the primary author using content analysis to explore the feasibility, fidelity and perceived acceptability. To ensure the accuracy and reliability of the data analysis, a rigorous process will be followed. To begin with, half of the transcripts will undergo double coding by two independent researchers. The remaining transcripts will be coded by AS alone. In case of any discrepancies in the coding, the researchers will discuss and arrive at a consensus.

During the evaluation design phase, reported outcomes of previous farm interventions were considered while determining the factors for measuring the programme’s success. For example, farm safety literature suggests that 25–77% of the farmers contacted could be expected to agree to participate [63–67], and an average of 80–100% satisfaction was reported among the participants with the previous farm safety intervention programmes [9, 67, 68]. Since the study will be conducted in the summer, farmers’ busy work schedules and vacation plans are also expected to influence participation and retention [69]. To measure treatment fidelity, the study will adhere to the National Institutes of Health Behavior Change Consortium guidelines, which define high treatment fidelity as having more than 80% adherence to the intervention checklist [49]. The researchers will use the intervention checklist, which provides a clear outline of the key components of the intervention and serves as a guide for implementation. The researchers will evaluate adherence by comparing the actual implementation of the intervention to the checklist and assessing the percentage of key components that were successfully implemented. The criteria outlined in Table 1 are established a priori to evaluate the success of the study, specifically the feasibility of implementing the intervention, the extent to which participants received the intended intervention and the acceptability of the intervention among participants. If some of the criteria are not met, the authors will investigate the potential causes of failures and consider changes to the intervention components and its delivery methods before deciding whether to recommend the development of a future larger trial to test the effectiveness of the intervention.

### Data management and monitoring

All participants will be assigned an identification number and a pseudonym, which will be used for the discussion of transcripts by the research team and in the publication of the study results. Consent forms and audio/video/electronic recordings could all contain information that could be used to identify a participant. Audio and video recordings will be deleted after transcribing them. Forms and transcribed data will be retained for 7 years. Hard copies, if any, will be stored in a locked filing cabinet within a restricted area. Access to this filing cabinet will be restricted to the study personnel and will be overseen by the principal investigator. Soft copies and Electronic recordings of meetings will be password protected and will be stored on a OneDrive/SharePoint network of the research sponsor. Access to these files will be overseen by the principal investigator. Due to the low risks associated with this behavioural intervention, there are no pre-specified interim analyses or stopping rules. Due to the low risks associated with this behavioural intervention, the study anticipates minimal occurrence of adverse events directly associated with the intervention. Any adverse events will be reviewed by the study team according to the Teagasc Advisory Discussion Group safety guidelines.

## Discussion

This paper describes the protocol for a feasibility study that explores the feasibility, fidelity and acceptability of a novel intervention to address blind spots of tractors.

Evidence indicates that behavioural interventions have the potential to address the health habits of the population at a low cost [70]. Yet, the active adoption of behavioural change techniques in farm research has been slow. This study will provide useful information that will aid in adopting various BCTs to increase the knowledge, intention to adopt and adherence to preventive farm practices. Previous reviews and our own review of the farm safety literature identified a gap in theory-driven intervention development as well as the reporting and assessment of its active ingredients [1, 6, 7, 71]. The intervention content and the evaluation procedure are described in this study, which will enable the replication or adoption of the intervention or one of its ingredients in future studies.

## Conclusions

This study is limited, in generalisation, due to the small sample size. However, by evaluating intervention content and delivery, the study is expected to provide evidence of the feasibility of each ingredient, its delivery and its acceptance among participants. This could facilitate farm safety researchers to create and implement behavioural change theory-centred, tailored, and targeted strategies to address farm safety. 

## Supplementary Information

**Additional file 1. **Intervention breakdown and evaluation timeline

**Additional file 2. **BeSafe-Intervention Protocol

**Additional file 3. **Topic guide for introduction session

**Additional file 4. **Safety training procedure template

**Additional file 5. **Exit survey

**Additional file 6. **Topic guide for evaluation interview

**Additional file 7. **TiDier Checklist

**Additional file 8. **SPIRIT Checklist

## Data Availability

The dataset supporting the conclusions of this article is included within the article. Additional data supporting the project is available in the OSF repository.

## Abbreviations

Teagasc: The Irish Agriculture and Food Development Authority
BCT: Behaviour Change Techniques
BCW: Behaviour Change Wheel
COM-B: Capability, Opportunity, Motivation-Behaviour
DAFM: Department of Agriculture, Food and the Marine
HAS: Irish Health and Safety Authority
NFS: National Farm Survey
SPIRIT: Standard Protocol Items: Recommendations for Interventional Trials
TIDieR: The Template for Intervention DEscription and Replication
TFA: Theoretical framework of acceptability
