# Supplementary material for: Assessing the feasibility, fidelity and acceptability of a behaviour change intervention to improve tractor safety on farms: protocol for the BeSafe tractor safety feasibility study
Source: Pilot Feasibility Stud. 2023 Jul 4;9:114. doi: 10.1186/s40814-023-01319-w (PMC10318716; doi:10.1186/s40814-023-01319-w)
Supplement: Supplementary file 1 — Additional file 1. Intervention breakdown and evaluation timeline [file 40814_2023_1319_MOESM1_ESM.pdf]

Additional file 1 Detailed Intervention breakdown and Evaluation timeline

Target Behaviours

Target Behaviour	Who	What	When	Where
Demonstrate blind spots of tractors to family members/co-workers on their farm	Participant farmer	Give an on-field demonstration to a non-participant farmer/family member/neighbours	Within one week of the peer to peer demo*	Home/Farm field
Mark the zone of visibility around their tractor in a parking	Participant farmer	Mark the zone of visibility with a marker around a tractor	Within one week of the peer to peer demo*	Home/Farm field
Walk around the tractor before moving it from the parking area to ensure that nobody is near the tractor and no obstacles are present near-by	Participant farmer	Do a 360 degree check around the tractor	Everyday; Before taking out the tractor from the parking area	Around the tractor

** tentative time period*

Pre-Intervention interview

Tasks	Estimated time	When	Mode	Facilitators
Pre-Intervention 1-1 online Interview	15-20 minutes	1-7 days before the Demo	Telephone/Skype / Zoom/Team	Aswathi Surendran

Pre Demo activities to be completed by the research team

- ✓ Secure the event site perimeter
- ✓ Make sure the risk assessment and safety checklist is completed
- ✓ Set up an area for the facilitated discussion session next to demo site
- ✓ Test run of the peer to peer demo
- ✓ Test run of the facilitated discussion and completion of safety training procedure

Half-day in-person session

#	Tasks	Subtasks	Time (In minutes)	Primary Facilitator*	Does the activity has evaluation component?
1	Welcome & Introduction		10	Aswathi Surendran	No
2	BeSafe Introduction	Interactive discussion: The prevalence and impact of blind spot related accidents	10	Aswathi Surendran	No

3	Impact of blind spots	Interactive discussion: Estimating the distance travelled by the tractor in 3 sec (in a yard and on regular speed)	10	Aswathi Surendran	No
4	Peer to peer demo	Invite one of the participant(P1) to sit inside the parked tractor	30	Aswathi Surendran	No
		Invite 2 (P2, P3) other participants to perform the demo.			
		Ask P2 to position the kid sized cut-out at different spots around demo tractor.			
		Ask P1 to confirm whether the cut-out is visible or not.			
		P3 mark the areas that was not visible to the P1 using spray paint/chalk			
		Mark the non-visibility area around the tractor using spray paint/chalk			
		Take a picture of the tractor and the marked area			
		Repeat the task on a different tractor with an implement mounted with a different group of participants			
	Break		5		
5	Facilitated discussion	Explore who would be most benefitted in his family when participants demonstrates blind spots at his farm	30	Aswathi Surendran	
		Explore various strategies to conduct the demo that's suitable for each participant, including barriers and facilitators			Yes
		Explore ways to set up the visibility zone in his own designated parking area in his farm based on the type, size and location			
6	Safety training procedure	Complete a tailored document for each participant based on the input from facilitated discussion.	20	Aswathi Surendran	
		Rate their confidence on completing the activity.			Yes
		Participant and a peer who acts as a witness sign the contract			
7	Conclusion	Debriefing session	15	Aswathi Surendran	
		Exit poll			Yes
		Distribute the vouchers and materials to perform the demo and setup the visibility zone in own parking area.			

*Teagasc safety advisor will be present.

Post Demo activities to be completed by the research team

✓ Clean up the event site after the demo

Evaluation Phase

SMS Survey

SMS Survey	Estimated time	When	Mode	Facilitators
SMS survey	5 minutes	Based on participant's convenience	Online SMS based survey	Aswathi Surendran

Evaluation session

Tasks	Estimated time	When	Mode	Facilitators
Post-Intervention one-on-one online Interview	45-60 minutes	10-20 days after the event	Telephone/Skype / Zoom	Aswathi Surendran
