# Supplementary material for: Assessing the feasibility, fidelity and acceptability of a behaviour change intervention to improve tractor safety on farms: protocol for the BeSafe tractor safety feasibility study
Source: Pilot Feasibility Stud. 2023 Jul 4;9:114. doi: 10.1186/s40814-023-01319-w (PMC10318716; doi:10.1186/s40814-023-01319-w)
Supplement: Supplementary file 2 — Additional file 2. BeSafe-Intervention Protocol [file 40814_2023_1319_MOESM2_ESM.pdf]

Additional file 2 Intervention components and BCTs

BCT *	Tasks
1.1 Goal setting (behaviour) *	Safety training procedure: 1.1 Agreement to do a demo of the blind spots and an action plan for setting the demo<br>1.4 Agreement to park in the designated parking sport daily<br>1.6 Agreement to walk around the tractor and check perimeter before moving out of the parking area
1.2 Problem solving*	Brainstorming session:<br>2. Explore various strategies to conduct the demo that's suitable for each participant<br>3. Explore ways to set up the visibility zone in his own designated parking area in his farm based on the type, size and location
1.3 Goal setting (outcome)*	Safety training procedure:<br>1.2 Action plan for setting up the visibility zone and agreement to share the picture with research team
1.4 Action planning*	Safety training procedure:<br>1.2 Action plan for setting up the visibility zone and agreement to share the picture with research team<br>1.3 Strategies for parking the tractor in the designated area after work<br>1.5 Strategies to walk around the tractor before starting or moving it from the designated area
1.8 Behavioral contract*	Safety training procedure: 4. Participant and a peer who acted as a witness sign the contract
1.9 Commitment	Safety training procedure:<br>1.1 Agreement to do a demo of the blind spots and an action plan for setting the demo<br>1.2 Action plan for setting up the visibility zone and agreement to share the picture with research team<br>1.4 Agreement to park in the designated parking sport daily<br>1.6 Agreement to walk around the tractor and check perimeter before moving out of the parking area<br>4. Participant and a peer who acted as a witness sign the contract
3.1 Social support (unspecified)	Safety training procedure: 4. Participant and a peer who acted as a witness sign the contract

3.2 Social support (practical)	Facilitated discussion:<br>2. Explore various strategies to conduct the demo that's suitable for each participant<br>3. Explore ways to set up the visibility zone in his own designated parking area in his farm based on the type, size and location
4.1 Instruction on how to perform a behaviour	Demonstration of the blind spots
5.1 Information about health consequenc es	Demonstration:<br>1. One of the farmers sit on a tractor and ask other farmers to stand around<br>2. Ask that farmer to locate others while sitting on the tractor and later walking around the tractor<br>3. Repeat this step with non-driver participants standing at various distances from the tractor and with people/cut out of children of different heights. A strong focus on stating that these cut-outs/people represent their family members
5.2 Salience of consequenc es *	Demonstration:<br>3. Repeat this step with non-driver participants standing at various distances from the tractor and with people/cut out of children of different heights. A strong focus on stating that these cut-outs/people represent their family members
6.1 Demonstrat ion of the behaviour	Demonstration of the blind spots
8.1 Behavioral practice/reh earsal*	Demonstration of the blind spots
8.3 Habit formation	Safety training procedure
9.3 Comparativ e imagining of future outcomes*	Demonstration:<br>3. Repeat this step with non-driver participants standing at various distances from the tractor and with people/cut out of children of different heights. A strong focus on stating that these cut-outs/people represent their family members

12.1 Restructuring the physical environment	Brainstorming session:3. Explore ways to set up the visibility zone in his own designated parking area in his farm based on the type, size and location<br>Safety training procedure
12.5 Adding objects to the environment	1. Provide the marker or similar items to setup the visibility zone<br>2. Provide materials for demonstration at home
13.1 Identification of self as role model*	Safety training procedure: 1.1 Agreement to do a demo of the blind spots and an action plan for setting the demo

*Identified as active ingredient
