# Supplementary material for: Assessing the feasibility, fidelity and acceptability of a behaviour change intervention to improve tractor safety on farms: protocol for the BeSafe tractor safety feasibility study
Source: Pilot Feasibility Stud. 2023 Jul 4;9:114. doi: 10.1186/s40814-023-01319-w (PMC10318716; doi:10.1186/s40814-023-01319-w)
Supplement: Supplementary file 3 — Additional file 3. Topic guide for introduction session [file 40814_2023_1319_MOESM3_ESM.pdf]

Additional file 2 Topic guide for introduction session

Please find below the topic guide for the audio/video-based online introduction session with the participants. The virtual one-on-one session will be scheduled 1-7 days before the intervention based on participant convenience. The objective of this semi structured call is to:

1. To clarify the questions farmers have regarding the project
2. Collect the consent for the program if they haven't already
3. Collect the demographic data
4. Enquire if they have any special needs for the demonstration day (hearing/visual aids, dietary restrictions, etc.)
5. Create a rapport with the participants

Estimated duration: 15-20 minutes

<u>Topic Guide</u>

Briefing:

1) Thank the participant for agreeing to take part.
2) Introduce self.
3) In this study, we will be introduce a safety program that we have developed to a selected few Irish farmers and collect their feedback.
4) If at any time during this call, you do not wish to answer a question that is okay.
5) I would like to record our conversation. The recording will be typed out, but everything you say will be anonymous. Your name and any names or places you mention will be taken out, so that if someone read your interview they would not know who you are.
6) If, at any stage, you wish to stop the audio recorder, please let me know.

7) Do you have any questions?

Topics to be explored: *Below is a list of questions to be discussed in this study. The work will remain flexible with respect to participants' agendas but we will cover the main topics outlined below.*

1. We would like to collect the demograpgic data first. May I know your age?
2. What type of farm do you have?
3. Do you work part time or full time? On an, average how many hours do you work in a day/week?
4. How many tractors do you have? Do you have any implements?
5. How many of those tractors have the following features?
   - ✓ Roll over protective frame
   - ✓ Power take off (PTO) master shield/output guard
   - ✓ Neutral start switch *
   - ✓ Hazard alert symbol or other safety signs
   - ✓ How many have roll back protection
   - ✓ Reverse assistance mirror
   - ✓ Reverse assistance camera
   - ✓ Lights
   - ✓ Horn
   - ✓ Reversing beeper

6. Is your family involved in the farm activities? If yes, how do they assist you on the farm?
7. Do you have contractors or other workers to assist you on the farm?
8. Have you or your loved ones ever been in a farm accident involving machine or equipment?

9. We would be providing a light snack and beverage on the in-person event day. Do you have any dietary restrictions that we should know?
