# Supplementary material for: Assessing the feasibility, fidelity and acceptability of a behaviour change intervention to improve tractor safety on farms: protocol for the BeSafe tractor safety feasibility study
Source: Pilot Feasibility Stud. 2023 Jul 4;9:114. doi: 10.1186/s40814-023-01319-w (PMC10318716; doi:10.1186/s40814-023-01319-w)

Additional file 4: Safety training procedure template

BeSafe-Tractor

Name :………………………….

Demo #:………………………..   Date :……………………………

Would you like to take the changes we discussed today to your home and farm?
The plan outlined in this plan will guide and assist you in improving the safety on your farm and raising awareness about the risks among family members and co-workers.

Short term goals:

1. *I will identify a general parking space on my farm and demonstrate blind spots of the tractors and implements to my family/co-workers.. Encourage your attendee to estimate the distance covered by tractor in 3 secs.*

When will I likely complete this goal (Preferred date)?

To whom would I like to demonstrate blind spots (Eg: family members/employees /neighours/discussion group members/students)?

How confident am I that I will do this? ____________ (on a scale of 0 to 10, with 0 being not at all confident and 10 being completely confident)

2. *I will set up a no-visibility zone to raise the awareness among your audience and document the measurements.*

When will I likely complete this goal (Preferred date)?

How confident am I that I will do this? ____________ (on a scale of 0 to 10, with 0 being not at all confident and 10 being completely confident)

Everyday Goals:

3. *Every day, before starting or moving the tractor from the parking area, I will walk around the tractor to ensure that no person or obstacle is present near-by*

How confident am I that I will do this? ____________ (on a scale of 0 to 10, with 0 being not at all confident and 10 being completely confident)

List possible obstacles to achieving your safety goals

List possible solution to overcome the obstacles

List possible strategies to perform the safety goals and address blind spots

I, ____________________________, will ensure that the safety goals are carried out to ensure the safety of my family, co-workers and me.

My friends who co-signed the safety training procedure, witnessed and assisted me in coming up with this plan.

____

____

Your Signature
Signature of the peer
Date

I would like to receive an SMS survey to report the progress of the goals on the following date and time:

On ………………………………… At ……………………………….. (Please provide your preferred time to receive the SMS survey)

I would like to participate in the online interview session and share my feedback on the following date and time:

On ………………………………… At ……………………………….. (Please provide your preferred time to schedule the interview)

Thank you for participating in the BeSafe session!

1. *You can measure and mark the no-visibility zone of your tractor at your farm using this template.*

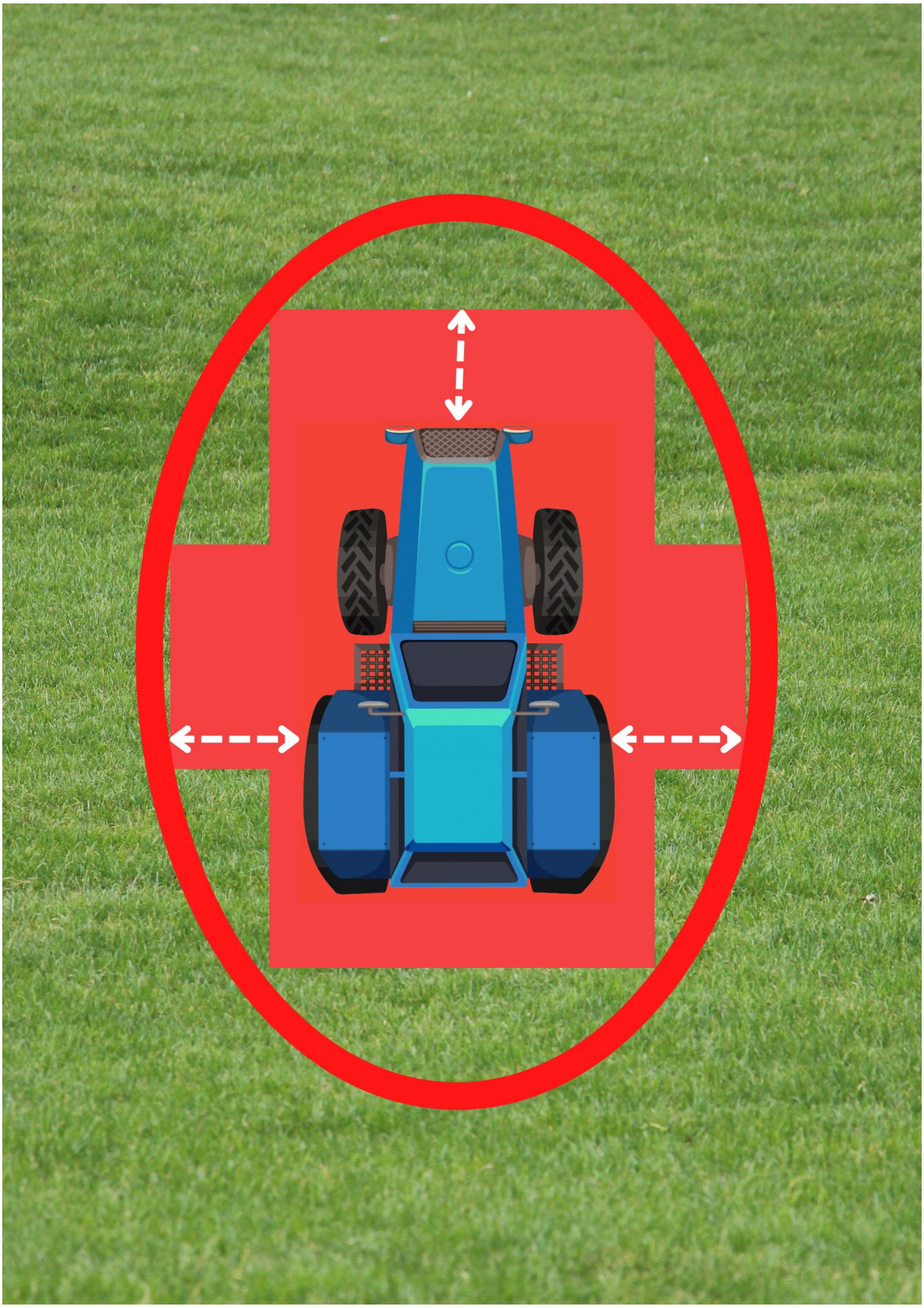

Supplement: Supplementary file 4 — Additional file 4. Safety training procedure template [file 40814_2023_1319_MOESM4_ESM.pdf]
