# Supplementary material for: Assessing the feasibility, fidelity and acceptability of a behaviour change intervention to improve tractor safety on farms: protocol for the BeSafe tractor safety feasibility study
Source: Pilot Feasibility Stud. 2023 Jul 4;9:114. doi: 10.1186/s40814-023-01319-w (PMC10318716; doi:10.1186/s40814-023-01319-w)
Supplement: Supplementary file 5 — Additional file 5. Exit survey [file 40814_2023_1319_MOESM5_ESM.pdf]

Additional file 4 Exit survey questions

Please find below the tentative questions and template for the exit poll survey. Objective of this survey is to gain the immediate feedback from the participants, evaluate the acceptability among participants who drop out before the post-intervention interview and set-up the slot for the post-intervention interview. Participants would be encouraged to fill out the form and drop it in a box to maintain the anonymity.

Participant Satisfaction Exit Survey Questionnaire

Demo # Survey # Date :

1. **How do you appreciate various aspects of the program?**

	**Very Useful**	**Useful**	**Neutral**	**Not Useful**
**Peer to Peer demo**				
**Facilitated discussion**				
**Safety training procedure**				

2. **Please indicate your level of agreement or disagreement with each of these statements regarding the program. Place an "X" mark in the box of your answer.**

**Statements**	**Strongly Agree**	**Agree**	**Neutral**	**Disagree**	**Strongly Disagree**
**The program met my expectations regarding what I wanted to learn.**					
**I obtained a clearer understanding of the following topic(s) demonstrated:**					

Demonstration of Blind spots					
Setting up visibility zone					
Checking the perimeter of the tractor					
**I think the day was well structured.**					
**I will apply what I learned today at home**					
**I will recommend this program to my friends**					

May we contact you in approximately <<# days> for a telephone interview?

If yes, preferred data, time and mode of contact?

Do you have any other suggestions?

Thank you for participating in the BeSafe session!
