# Supplementary material for: Assessing the feasibility, fidelity and acceptability of a behaviour change intervention to improve tractor safety on farms: protocol for the BeSafe tractor safety feasibility study
Source: Pilot Feasibility Stud. 2023 Jul 4;9:114. doi: 10.1186/s40814-023-01319-w (PMC10318716; doi:10.1186/s40814-023-01319-w)
Supplement: Supplementary file 6 — Additional file 6. Topic guide for evaluation interview [file 40814_2023_1319_MOESM6_ESM.pdf]

Additional file 6 Topic guide for evaluation interview

Please find below the topic guide for the post-intervention audio/video interview with the participants. The evaluation interview will be scheduled 7-20 days after the intervention based on participant convenience. The objective of this semi-structured interview is to:

1. To assess the acceptability of the program and its delivery among participants
2. To assess the acceptability of each active ingredient and its delivery among participants
3. Assess the fidelity sub-constructs (receipt of treatment & enactment of treatment skills)

Estimated duration: 45-60 minutes

Topic Guide
Briefing:

1) Thank the participant for agreeing to take part.
2) Introduce the objective of this call
4) If at any time during this call, you do not wish to answer a question that is okay.
5) I would like to record our conversation. The recording will be typed out, but everything you say will be anonymous. Your name and any names or places you mention will be taken out, so that if someone read your interview they would not know who you are.
6) If, at any stage, you wish to stop the audio recorder, please let me know.
7) Do you have any questions?

Topics to be explored: *Below is a list of questions to be discussed in this study. The work will remain flexible with respect to participants' agendas but we will cover the main topics outlined below.*

1. Tell me about your experience with the peer to peer demonstration: Was it useful? why?
   - ✓ Do you think now you can teach others about blind spots? If so, will you follow the same style of demonstration? If not, what changes?
   - ✓ Does practising and rehearsing with the peers helped? Yes/No, Why?
   - ✓ Was it difficult to imagine the family members in the blind spot? Why?
   - ✓ Do you think better understanding and setting up the visibility zone improved awareness about immediate environment? Does it help to protect the family members?
   - ✓
2. Do you think elder farmers teaching younger ones in similar demo sessions would encourage them to adopt safer habits as you?
   - ✓ Do you think demonstrating the blind spot helped your family/others to become more aware of blind spots?
   - ✓ Why do you think it was effective/not effective?
   - ✓ Do you think your family members and young farmers look up to the way you work?

3. Tell me about your experience with the brainstorming session:
   - ✓ Does sitting with your peers helped to identify the barriers and find the solutions?
   - ✓ Do you had the feeling that you could share your own knowledge and experience ?

4. Tell me about your experience with creating personalized safety training procedure:
   - ✓ Was it easy to create and understand the protocol?
   - ✓ Was personalizing it made it more beneficial?
   - ✓ Were the goals practical and efficient?
   - ✓ Did planning the task step by step and setting up the goals helped you to complete the tasks at home?

5. What do you think about creating a protocol and co-signing it with your peers?
   - ✓ Was it useful?

- ✓ Were you comfortable co-signing your own protocol and your peer's protocol?

6. Did you complete the following task that we agreed upon in the demo and co-signed with your peer farmer?
    - ✓ Demonstration of the blind spot to family/others
    - ✓ Setting up the visibility zone
    - ✓ Measure the distance (tractor to visibility zone border)
    - ✓ Do you walk around the tractor and check the perimeter before moving it from the parking area?
    - ✓ If not, why?
    - ✓ Did you face any unexpected difficulty while demonstrating blind spots to family/others and setting up the visibility zone?

7. Did you talk to other people about this demonstration? Would you recommend this program to a friend or neighbour?
8. Have you attended another demonstration event during the intervention period?
9. How helpful was the materials distributed during the workshop to complete tasks at home? What other resources were required?
10. We talked about various techniques we had for the workshop. Do you think similar techniques can be used to address other safety concerns? Which techniques would be effective? What safety concerns can be addressed?
11. Have you thought about making/already made any changes in your farm as a result of participation in the peer to peer demonstration? For e.g.: safety devices or reversing assistance devices like mirrors, camera
12. Did the workshop event helped you to gain knowledge or skills? If so,

- ✓ Which tasks?
- ✓ What knowledge/skills improved?
- ✓ Do you think demonstrating the blind spot helped your family/others to become more aware of blind spots?
- ✓ Is there anything else that you think would be valuable to improve the program?

13. Suggestions
    - ✓ If you had a chance to give advice to the facilitators of this program, what advice would you give?
    - ✓ Did facilitators failed to address any concerns related this topic?

14. Summary

- ✓ Was there anything I left out?
- ✓ Anything else you would like to tell me
