# Supplementary material for: Assessing the feasibility, fidelity and acceptability of a behaviour change intervention to improve tractor safety on farms: protocol for the BeSafe tractor safety feasibility study
Source: Pilot Feasibility Stud. 2023 Jul 4;9:114. doi: 10.1186/s40814-023-01319-w (PMC10318716; doi:10.1186/s40814-023-01319-w)
Supplement: Supplementary file 7 — Additional file 7. TiDier Checklist [file 40814_2023_1319_MOESM7_ESM.pdf]

Intervention description based on TIDieR checklist[1]

Article: Assessing the feasibility, fidelity and acceptability of a behaviour change based intervention to reduce tractor related accidents on farms: protocol for the BeSafe feasibility study.

Author(s): Ms. Aswathi Surendran[1], Dr Jenny McSharry[1], Dr David Meredith[2], Dr John McNamara[2], Mr Francis Bligh[2], Dr Denis O'Hora[1]
[1]School of Psychology, National University of Galway, Ireland
[2]TEAGASC, Ireland

Name	BeSafe intervention to reduce tractor related accidents on farms
Why? (rationale)	Described above in the background section.
What? (procedure)	TEAGASC will make the primary contact to contact the farmers discussion groups. Once the group indicate their interest, advisors send invitation letters, participant information sheet and consent form to the farmers. On week 1, the participants will receive a call from the primary facilitator (AS) to collect the demogrphy information and detail the project. In the next phase, participants would be invited to participate in the demo session facilitatied by the primary facilitator. Demo would provide paticipants with strategies to address blind spots on farm and personalized safety training procedure with safety goals to ccomplet at their own homes.Subsequently a SMS would be send to track the progress in completing their goals. A telephone intreview will be set up after 2 weeks of the demo session to collect the feedbcak of the participants<br><br>The breakdown of the tasks along with estimated time and facilitator details are provided in the Appendix section (Intervention & Evaluation timeline)
What? (Materials)	Participants will receive:<br>A tailored safety training procedure<br>Materials to perform the demo and setup the visibility zone in own parking area
Who provided?	Two researchers (AS, DOH) will deliver the intervention on selected Teagsc campuses.<br>Teasgsc farm safety experts will be present on the demo location during the demo session to ensure the safety of the participants
How?	Face-to-face in a group setting:<br>The intervention consist of peer to peer demo of blind spots of tractors, discussion and demonstration of strategies to address blind spots and investigation of individual barriers to implementing these strategies. The safety training procedure with pre-determined goals will be tailored to suit the participant's farm and barriers discussed in the discussion session. Participants would be encouraged to complete the goals through an SMS survey.

[1] Hoffmann TC, Glasziou PP, Boutron I, Milne R, Perera R, Moher D, et al. Better reporting of interventions: template for intervention description and replication (TIDieR) checklist and guide. Bmj [Internet]. 2014;348. Available from: https://www.bmj.com/content/348/bmj.g1687

Where?	The intervention will take place on a farm field selected and approved by Teagasc
When and how much?	The communication will be initiated once the potential participant indicate their interest to Teagasc advisor. The face to face demo session will be approximately 3.5 hours. The demo session would be running once a week for 4 weeks. Participants can attend one of these sessions based on their convenience.
Tailoring	The safety training procedure will be tailored to individual needs of the participants, based on their farm setting, resources available and preferences.
How well? (adherence/fidelity)	The facilitators will be audio recording the entire session and making observational notes. Fidelity will be measured using a pre-established checklist created based on the study design, purpose and fidelity framework for behaviour change research by analyzing the recorded data.

[1] Hoffmann TC, Glasziou PP, Boutron I, Milne R, Perera R, Moher D, et al. Better reporting of interventions: template for intervention description and replication (TIDieR) checklist and guide. Bmj [Internet]. 2014;348. Available from: https://www.bmj.com/content/348/bmj.g1687
